# Dual Antibacterial and Soft-Tissue-Integrative Effect of Combined Strontium Acetate and Silver Nitrate on Peri-Implant Environment: Insights from Multispecies Biofilms and a 3D Coculture Model

**DOI:** 10.1021/acsami.5c01093

**Published:** 2025-04-22

**Authors:** Marjan Kheirmand-Parizi, Katharina Doll-Nikutta, Carina Mikolai, Dagmar Wirth, Henning Menzel, Meike Stiesch

**Affiliations:** † Department of Prosthetic Dentistry and Biomedical Materials Science, Hannover Medical School, Carl-Neuberg-Strasse 1, 30625 Hannover, Germany; ‡ Lower Saxony Center for Biomedical Engineering, Implant Research and Development (NIFE), Stadtfelddamm 34, 30625 Hannover, Germany; § Helmholtz Centre for Infection Research, 38124 Braunschweig, Germany; ∥ Institute for Technical Chemistry, Braunschweig University of Technology, Hagenring 30, 38106 Braunschweig, Germany

**Keywords:** silver, strontium, peri-implant, soft tissue seal, antibacterial, 3D *in vitro* model

## Abstract

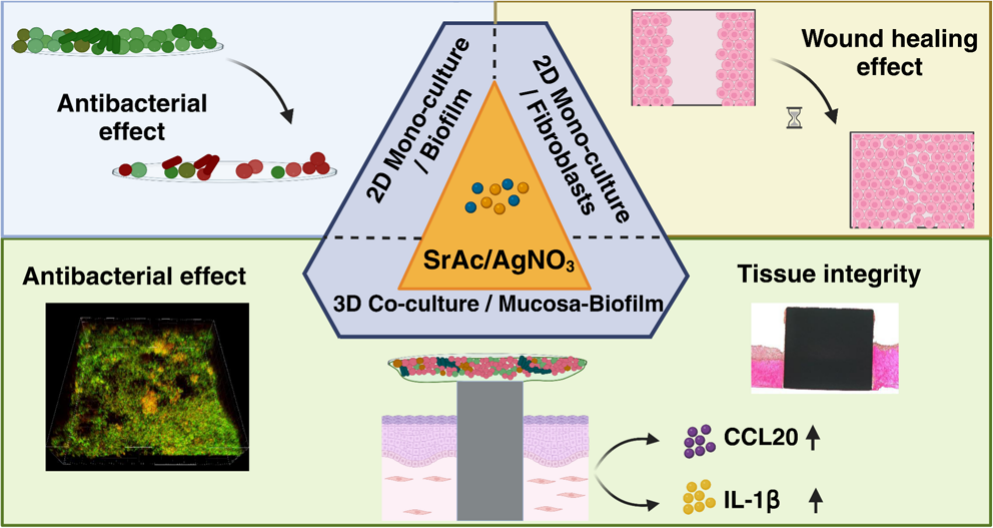

Creation of a biological seal and efficient antibacterial qualities in the peri-implant environment is essential for the success of dental implants. Therefore, novel multifunctional strategies are being developed to address these issues, aiming at the simultaneous improvement of tissue integration and hindering pathological biofilm formation. In this study, we investigated the effect of tissue-promotive strontium acetate (SrAc), antibacterial silver nitrate (AgNO_3_), and their combination on oral soft tissue cells and an oral multispecies biofilm not only in monoculture setups but also in a three-dimensional (3D) implant-tissue-oral bacterial-biofilm model (INTERbACT model) that takes the naturally occurring interactions into account. Application of SrAc led to improved fibroblast migration in the monoculture setting, without impairment of metabolic activity, even upon additional AgNO_3_ administration. Notably, the combined treatment of SrAc and AgNO_3_ resulted in a synergistic antibacterial effect during biofilm formation as well as on early matured biofilms. Most interestingly, the antibacterial effect of the combined treatment was even further enhanced within the coculture setup leading to increased bacterial death and decreased biofilm volume. The 3D tissue in the coculture setup underwent the combined treatment with a notable rise in CCL20 and IL-1β levels. Histologically, only the AgNO_3_-treated groups exhibited damage to the integrity of the epithelial barrier. Therefore, the results of this study demonstrated promising dual antibacterial and tissue-integrative characteristics of combined AgNO_3_ and SrAc in the dental implant environment. Additionally, the study emphasizes the importance of considering naturally occurring tissue–bacteria interactions for reliable *in vitro* testing of novel implant materials.

## Introduction

The gingival mucosa naturally safeguards the periodontal tissues of teeth by forming a protective seal that prevents bacterial penetration.^1^ Moreover, the epithelium layer is capable of reacting to external stimuli by synthesizing a number of chemokines and cytokines, further enhancing its defensive capabilities against microbial invasion.^1,2^ However, around dental implants, the soft tissue seal is compromised. Connective tissue at the implant surface lacks an adequate number of fibroblasts and perpendicular collagen fibers, which enhances the risk of bacterial infiltration, impaired tissue integration, and potential complications such as peri-implantitis or peri-implant mucositis.^3,4^ Bacterial infection induces an inflammatory response, which can result in the destruction of the peri-implant tissues (hard and soft tissues) and in severe cases even implant loss.^3^ According to previous systematic reviews, 63.4 and 18.8–26.0% of the patients with implant function time for more than 5 years were diagnosed with peri-implant mucositis and peri-implantitis, respectively.^5,6^ Research has demonstrated that the success of dental implants does not only depend on the integration of bone to the implant surface, but primarily on creating a peri-implant soft tissue barrier to protect the implant and hard tissue below from bacterial invasion.^7,8^ Consequently, the long-term success of dental implants relies on the simultaneous combination of effective antibacterial properties in the peri-implant environment and the formation of biological seal around the implant.^8,9^

Recently, implant materials incorporating bioactive ions have shown to be a promising approach to address these challenges.^10,11^ Among these ions, a potential double benefit of Ag ions (Ag^+^) as well as Sr ions (Sr^2+^) as antibacterial and tissue-integrative agents could be achieved.^10−12^ In our previous systematic review, Sr/Ag-based titanium coatings showed enhanced osteogenic and antibacterial effects suggesting a potential synergistic effect of this combination.^13^ Ag ions exhibit strong antimicrobial properties by disrupting the bacterial cell wall/membrane, damaging the signal transduction pathways, and causing oxidative stress within the cells.^14^ Numerous studies have demonstrated that Ag-modified titanium surfaces can reduce the growth of various bacteria commonly associated with peri-implant infections.^15,16^ However, Ag has been mostly studied against planktonic bacteria rather than on bacterial biofilms. Nevertheless, these three-dimensional, matrix-enclosed bacterial agglomerates have higher tolerance against antibacterial agents and are more clinically relevant.^14,15^ On the other hand, Sr particularly in the form of Sr-ranelate, is well known for its osteogenic and bone formative effects both *in vitro* and *in vivo*.^17^*In vivo* studies have shown that Sr-modified titanium implants promote osseointegration of implants, bone apposition, bone-to-implant contact (BIC) around the implants enhancing the long-term stability.^18^ Recent studies have also shed light on the effects of Sr on gingival fibroblast cells. These studies have revealed that Sr can reduce apoptosis and increase proliferation, migration, and adhesion of gingival fibroblast cells, which play a crucial role in peri-implant mucosal healing.^19−21^ Moreover, some studies even showed a limited antimicrobial effect following Sr administration against oral bacteria associated with peri-implantitis.^12,22^ Therefore, a dual antibacterial and soft tissue integration of combined Sr/Ag treatment could be possible. However, to the best of our knowledge, no previous studies have addressed the effect of combined Sr/Ag treatment on both soft tissue healing and antibacterial properties simultaneously.

When the combined application of Ag and Sr on titanium implants has been investigated for its potential to inhibit bacterial growth and promote bone formation,^10,23^ most of these strategies used comparable simple testing platforms, such as single bacteria, single-layer cells, monocultures, and two-dimensional (2D) setups.^10,23,24^ However, while these methods facilitate comparisons across various *in vitro* research studies, their direct clinical translation is limited, as they lack critical native morphologies and interactions. For example, clinically grown bacteria form biofilms on implant surfaces including multispecies bacteria that are more pathogenic and resistant to antibacterial agents.^25^ Additionally, we previously observed a reduced antibacterial effect when AgNO_3_ and SrAc were used in a cell/bacteria coculture setup as compared to monoculture conditions.^12^ But also using a single-cell type cocultured with a single-bacterial species may lead to confounding results as they only represent interactions between selected host and microbe.^26^ 2D cell culture models are unable to reflect the complex characteristics of native tissue environments or facilitate the cell–cell and cell–matrix interactions that contribute to the interpretation of surrounding biochemical signals by the cells.^27,28^ Consequently, it is crucial to explore the antibacterial effects and tissue responses induced by chemical agents by employing *in vivo* or semi*in vivo* models in order to better understand the complex interactions between bacteria, chemicals, and tissues and to obtain more clinically relevant results. Particularly, three-dimensional (3D) models encompassing implant, soft tissue, and biofilm would be essential to thoroughly analyze the interactions between Sr/Ag and surrounding soft tissue as well as oral biofilm.^26,29^

The aim of this study was to comprehensively evaluate the antibacterial and soft tissue healing effects of SrAc/AgNO_3_ by considering clinically occurring interactions between oral cells and bacteria during the initial healing phase. For this purpose, first the effect on gingival fibroblasts was assessed in a 2D monoculture assay using a single-cell layer. In addition, the antibacterial effect was analyzed using an oral multispecies biofilm model. Following these evaluations, SrAc/AgNO_3_ treatment was then applied in a previously developed complex 3D implant-tissue-oral bacterial-biofilm model (INTERbACT) to assess how cell-bacteria interactions influence the dual antibacterial and soft-tissue-integrative effect. This will yield valuable insights into the multifaceted effects of SrAc/AgNO_3_ in the peri-implant environment and reveal features related to the evaluation of complex interactions in clinically relevant models when testing novel implant materials.

## Materials and Methods

### Chemicals

Strontium acetate (SrAc, Sr(CH_3_CO_2_)_2_) and silver nitrate (AgNO_3_, BioXtra, >99%) were purchased from Sigma-Aldrich (Merck KGaA, Darmstadt, Germany). Both chemicals were freshly dissolved prior to experiments in sterile Milli-Q water to obtain certain concentrations (SrAc: 100 mg/mL; AgNO_3_, 100 μg/mL). To reach the final concentration in experimental volume, the concentrations were further diluted accordingly: SrAc: 0.5 mg/mL, SrAc: 1 mg/mL, and AgNO_3_: 0.5 μg/mL.

### Cell Culture

Human gingival fibroblasts (HGFs, 1,210,412, Provitro GmbH, Berlin, Germany) were cultured in Dulbecco’s modified Eagle’s medium (DMEM, P04-04500, PAN-Biotech GmbH, Aidenbach, Germany) supplemented with 10% fetal bovine serum (FBS, P30-3309, PAN-Biotech GmbH) and 1% penicillin (P/S, Sigma-Aldrich). HGFs were used in passage 10. Immortalized human oral keratinocytes (OKF6/TERT-2) were cultured in KerSFM medium (10725-018, Gibco Life Technologies, U.K.) supplemented with 25 μg/mL bovine pituitary extract (BPE, Gibco Life Technologies, U.K.), 0.2 ng/mL human recombinant epithelial growth factor (EGF, Gibco Life Technologies, U.K.), 0.3 mM calcium chloride (CaCl_2_, PromoCell, Heidelberg, Germany), and 1% P/S. OKF6 cells were used in passage 25–35. Both cell lines were incubated at 37 °C in a 5% CO_2_ humidified atmosphere.

### Cell Viability Assay

CellTiter-Blue Cell Viability Assay (Promega, Madison, WI) was used to evaluate cell viability according to the manufacturer’s protocol. Briefly, a density of 1 × 10^5^ HGF cells/mL (100 μL/well) was seeded in a 96-well plate (CELLSTAR-Greiner Bio-One, Kremsmünster, Austria). After incubation at 37 °C in 5% CO_2_ for 24 h, the medium was replaced, and the cells were exposed to varying concentrations of chemicals in their respective medium (SrAc 0.5 mg/mL, SrAc 1 mg/mL, AgNO_3_ 0.5 μg/mL, SrAc 0.5 mg/mL + AgNO_3_ 0.5 μg/mL, SrAc 1 mg/mL + AgNO_3_ 0.5 μg/mL) for 24 h. Fluorescence units were measured upon the addition of CTB reagent after 4 h using a plate reader (Tecan, Infinite M200Pro, Männedof, Switzerland; excitation/emission: 560/590 nm). Three biological and two technical repetitions, each, were conducted.

### Wound Healing Assay for Cell Migration Analysis

HGF cells were seeded at 3 × 10 ^5^ cells/mL into a Culture-Insert 2 Well in μ-Dish 35 mm (ibidi GmbH, Gräfelfing, Germany) with 70 μL volume in each well. After 24 h (100% cell confluency), the silicon insert was gently removed using sterile tweezers, leaving a 500 ± 100 μm cell-free gap according to the producer‘s product details. Cells were washed twice with medium to remove nonadherent cells or cell debris. Dishes were incubated with fresh DMEM, with and without SrAc and AgNO_3_ stimulation at similar concentrations like for the cell viability assay. Three images of cellular migration into gaps were taken from each sample at 0, 9, 24, and 36 h using an optical microscope (Leica DMi1, Leica Microsystems, Mannheim, Germany) with a 10-fold magnification objective and LAS V4.8 software. The experiments were conducted in three biological and three technical repetitions, each. The percentage of cell-free gap area was measured in each image using the wound healing size tool in the ImageJ software (National Institutes of Health, Bethesda, MD). The average percentage of wound closure after 36 h was calculated using the formula: [(gap area at time 0) – (gap area at 36 h)/(gap area at time 0)] × 100. The process of wound closure was monitored to the point where the gap was fully closed, at least in one of the experimental groups.

### Immunofluorescence Staining

HGF cells were seeded with 5 × 10 ^4^ cells/mL cell density in a 12-well chamber, removable glass slide (ibidi GmbH) and 250 μL volume per well. After 24 h of cultivation, cells were fixed using 4% paraformaldehyde (PFA, Carl Roth GmbH, Karlsruhe, Germany) for 20 min at 4 °C. Permeabilization was applied using 0.1% Triton X-100 (T9284, Sigma-Aldrich) in phosphate-buffered saline (PBS, Sigma-Aldrich) for 10 min at room temperature. Following three times rinsing, fixed cells were treated with blocking solution of 2% BSA (bovine serum albumin, Sigma-Aldrich) for 30 min at 37 °C. In order to stain focal adhesion complexes, cells were incubated initially with primary antibody (Mouse Anti-Vinculin, 1:400, V9131, Sigma-Aldrich, overnight at 4 °C) and further with secondary antibody (Goat Anti-Mouse IgG Antibody, Cy3 conjugate, 1:200, Sigma-Aldrich, 1 h at room temperature). Actin cytoskeleton and nuclei were counterstained using Phalloidin-iFluor 488 (Abcam, Cambridge, U.K.) and 4′,6-diamidino-2-phenylindol (DAPI; Thermo Fisher Scientific) for 30 min at room temperature. Cells were visualized using a confocal laser scanning microscope (CLSM; Leica TCS SP8, Leica Microsystems, Mannheim, Germany) (laser line 488 nm, emission at 493–550 nm; laser line 552 nm, emission at 540–580 nm; and laser line 405 nm, emission at 350–470 nm). The Imaris software was used for three-dimensional reconstruction of stained cells.

### Bacterial Culture and Multispecies Biofilm (MSBF) Formation

*Streptococcus oralis* (ATCC 9811) was obtained from the American Type Culture Collection (Manassas, Virginia). *Actinomyces naeslundii* (DSM 43013), *Veillonella dispar* (DSM 20735), and *Porphyromonas gingivalis* (DSM 20709) were obtained from the German Collection of Microorganisms and Cell Cultures (Braunschweig, Germany). Bacteria were stored as glycerol stocks at −80 °C and precultivated in brain heart infusion medium (BHI; Oxoid Limited) supplemented with 10 μg/mL vitamin K (Carl Roth GmbH & Co. KG, Karlsruhe, Germany). Multispecies biofilms were formed as previously described.^30^ Briefly, equal volumes of the four different oral bacterial species were mixed to achieve an equal optical density at 600 nm of 0.01 per each bacterium and grown on glass coverslips (18 mm diameter, thickness 1, ThermoScientific Menzel) in BHI/vitamin K medium supplemented with 5 mg/L hemin under anaerobic conditions. Initially, chemicals (in certain concentration as previously described) were added to the medium during biofilm formation (0 h) and incubated for a duration of 24 h under anaerobic conditions. Following the determination of the chemical concentrations that affected biofilm formation, a subsequent experiment was conducted by adding the effective chemical concentrations after 24 h followed by 48 h of incubation time in anaerobic conditions.

### Biofilm Viability Assay

MSBF formed on coverslips were washed two times with phosphate-buffered saline (PBS, Sigma-Aldrich) and then incubated with 0.001% resazurin +10% biofilm medium (BHI/vitamin K/hemin) in PBS under anaerobic conditions at 37 °C. After 10 min, 100 μL of each well was transferred to a 96-well plate to measure the fluorescence units using a plate reader (Infinite M200Pro) within 530/590 nm excitation and emission wavelengths. A total of nine replicate experiments (three biological replicates and three technical replicates) were performed to validate the results.

### Biofilm Fluorescence Staining

After the metabolic activity was measured, the same MSBFs were fluorescently stained using SYTO9 and propidium iodide (LIVE/DEAD BacLight Bacterial Viability Kit, Thermo Fisher Scientific GmbH, Dreideich, Germany). A 1:1000 dilution of each stain was prepared in PBS and added to samples for 30 min of incubation in the dark. A 2.5% glutaraldehyde solution (Roth, Germany; 1:10 dilution with PBS) was then used to fix the samples. Biofilms were analyzed with a CLSM microscope using 488 and 552 nm laser lines and emission ranges from 500–550 to 650–750 nm for SYTO9 and propidium iodide, respectively. Five images with sizes of 387.5 μm × 387.5 μm and a z-step size of 5 μm were taken from each specimen in different positions. Quantification of live (SYTO9 stained), dead (propidium iodide stained), and total biofilm volume within each three-dimensional image was done using the Imaris x64 8.4 software package (Bitplane AG, Zurich, Switzerland). All experiments were performed in three biological and three technical replications, each.

### Testing Chemicals in a 3D Implant-Tissue-Oral Bacterial-Biofilm Model

As previously reported, an *in vitro* model of peri-implant mucosa with integrated implant and cocultured biofilm (INTERbACT model) was generated.^29^ In summary, the bovine collagen type-I hydrogel was used to embed HGFs. After 4 days, a precolonized titanium cylinder (machined surface, grade 4, 3 mm in diameter, 2.3 mm in height) was integrated into the HGF-collagen gel. After 1 week, OKF6 cells were seeded on top of the gel. Two days later, the tissues were placed in a liquid–air interface in order to stimulate stratification of epithelial cells for a further 2 weeks. In parallel, multispecies biofilm was formed as described above.^31^ For coculture, biofilms were placed upside down on top of titanium cylinders within the peri-implant mucosa model. Chemicals (SrAc 1 mg/mL, AgNO_3_ 0.5 μg/mL, SrAc 1 mg/mL + AgNO_3_ 0.5 μg/mL) were added gently to the coculture medium (inside and outside inserts) simultaneously. The coculture setup was incubated for 48 h at 37 °C in a 5% CO_2_ humidified atmosphere.

### Cytokine Expression, Histological and Microscopic Analysis of INTERbACT Model

Following coculture, the supernatants were collected and used to quantify cytokine expression using specific ELISA kits. CCL20 was quantified using the ELISA MAX Deluxe Set Human CCL20 (MIP-3α, Biolegend). IL-1beta was quantified using the Human IL-1β Mini ABTS ELISA Development Kit (PeproTech, Hamburg, Germany) and the TNF-α level was measured with the Human TNF-α Mini ABTS ELISA Development Kit (PeproTech). All ELISA kits were utilized following the manufacturer’s instructions. The concentrations of the respective cytokines were calculated using a four-parameter logistic (4-PL) equation obtained from the standard curve. According to the previous description, the peri-implant mucosa was histologically analyzed.^29^ In summary, tissues containing integrated implants were embedded in Technovit 9100 (Kulzer GmbH, Wehrheim, Germany), grounded to create slides with 22–36 μm thickness, and van Gieson stained. Sectioning, grinding, and histochemistry were performed at MORPHISTO GmbH, Offenbach am Main. Microscopic evaluation was done using a Zeiss Axioskop 40 microscope (Carl Zeiss GmbH, Jena, Germany). Moreover, Live/Dead staining of implant and peri-implant tissues was done using LIVE/DEAD BacLight staining, as described above. Fluorescently stained samples were visualized using CLSM (Leica TCS SP8) with 2.5× and 10× objectives and extinction and emission ranges as described. MSBFs were fluorescently stained and analyzed accordingly.

### Statistical Analysis

The data were statistically analyzed and graphically processed using GraphPad Prism Software 8.4 (GraphPad Software, Inc., La Jolla). Two-way analysis of variance (ANOVA) with Tukey’s multiple comparison correction was used to compare wound healing results in different groups at different time points. For all other results, the D’Agostino-Pearson test was employed to check for normal distribution. One-way ANOVA with Tukey’s multiple comparison correction was used for parametric data, and Kruskal–Wallis test was used for nonparametric data as stated in the respective results. For all comparisons, a significance level of α = 0.05 was defined.

## Results

### Effect of SrAc and AgNO_3_ on Cell Migration and Viability

**Figure 1:**
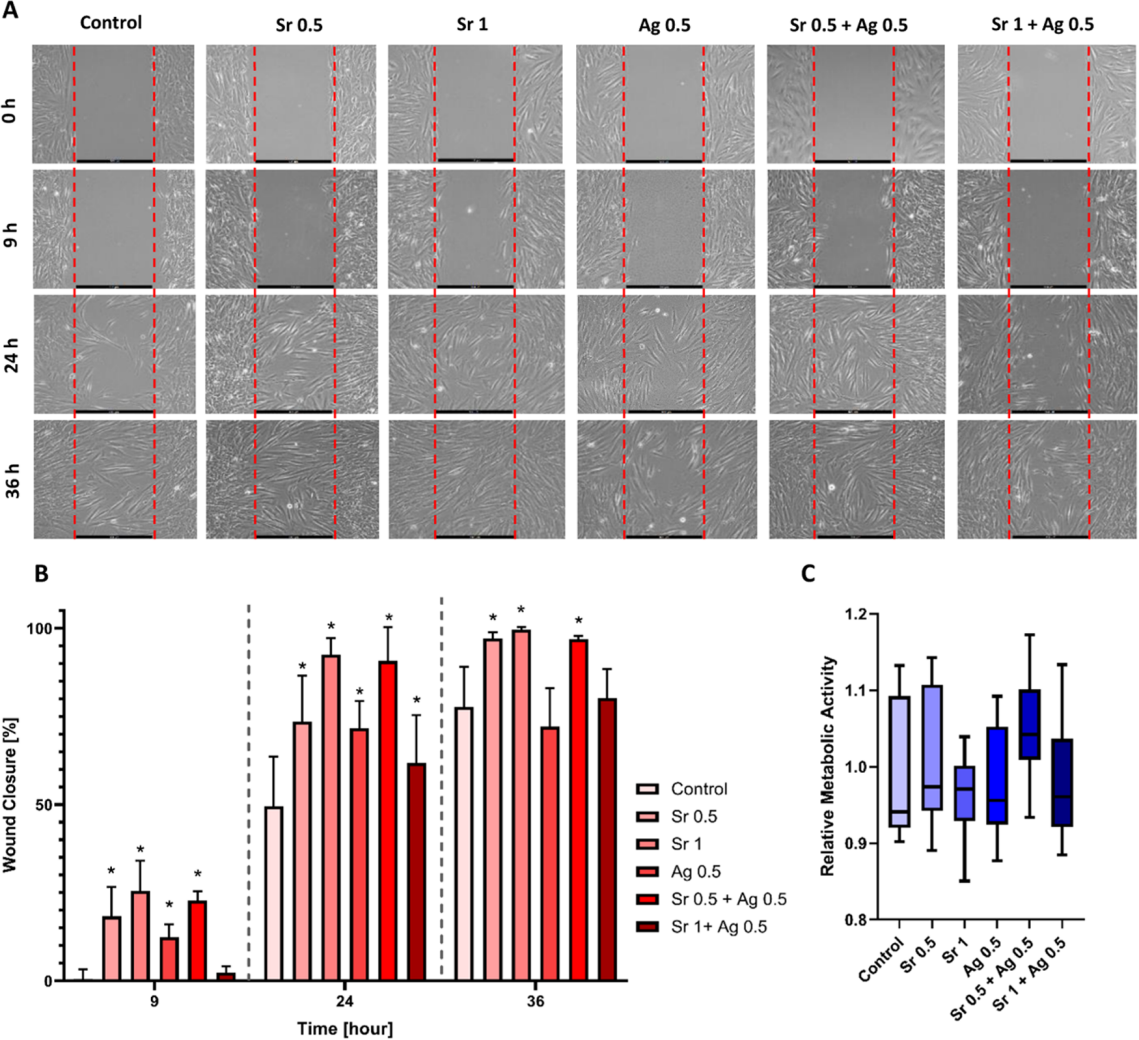
Effect of AgNO_3_ [μg/mL], SrAc [mg/mL] and their combination treatments on migration and viability of HGFs. (A) Representative photographs of cell migration in a standard wound closure assay after chemical treatment. Scale bars represent 500 μm. Gap surface area was quantitatively measured resulting in the data shown in (B) and *N* = 9. (C) Cell metabolic activity upon chemical treatment for 24 h relative to untreated cells determined by CellTiter-Blue assay and *N* = 6. Black stars indicate a statistically significant decrease compared to the control with *p* ≤ 0.05 at each time point. The significant differences were analyzed by a two-way ANOVA test for wound healing and Kruskal–Wallis test for CTB results.

To first assess the soft tissue healing effects of SrAc and AgNO_3_, a standard 2D monoculture wound healing assay was conducted to analyze the migration of fibroblasts. This assay is one of the basic methods used to study cell migration *in vitro*. It involves observing cell migration into a gap (wound) created on a monolayer of cells, which partially mimics *in vivo* cell migration.^32^ The migration of cells in control group (standard medium) was compared to cells treated with SrAc 0.5 mg/mL, SrAc 1 mg/mL, AgNO_3_ 0.5 μg/mL, SrAc 0.5 mg/mL + AgNO_3_ 0.5 μg/mL, and SrAc 1 mg/mL + AgNO_3_ 0.5 μg/mL for up to 36 h. Over time, cells gradually populated the gap, resulting in a final gap surface area of approximately 10% which equals an average closure rate of 77.6% within 36 h of observation in the control group (Figure 1A,B). A comparable closure rate was noted in the group treated with AgNO_3_ 0.5 μg/mL after 36 h of observation (72.15%). Interestingly, AgNO_3_ 0.5 μg/mL showed a statistically significant increase in wound closure after 9 h, but the effect was not further observed after 24 and 36 h. Both the SrAc 0.5 and 1 mg/mL treatments demonstrated significantly elevated migratory ability compared to the control group at all time points (9, 24, and 36 h), with the effect being more pronounced in the SrAc 1 mg/mL treatment. After 36 h, the average closure rates were 97.17% for SrAc 0.5 mg/mL and 100% for SrAc 1 mg/mL, respectively. While AgNO_3_ treatment accelerated wound closure without improving it, SrAc application not only accelerated wound closure but also improved it. The application of SrAc 0.5 mg/mL + AgNO_3_ 0.5 μg/mL as a combined treatment led to a statistically significant increase in cell migration and a decrease in gap size at all observed time points. In contrast, significant increase in cell migration was observed with SrAc 1 mg/mL + AgNO_3_ 0.5 μg/mL treatment only after 24 h of observation. The measured average closure rates after 36 h were 97% for SrAc 0.5 mg/mL + AgNO_3_ 0.5 μg/mL and 80.22% for SrAc 1 mg/mL + AgNO_3_ 0.5 μg/mL, respectively. While there were no significant differences in gap closure between SrAc 0.5 mg/mL alone and SrAc 0.5 mg/mL + AgNO_3_ 0.5 μg/mL after 36 h, there was a significant decrease in cell migration observed with SrAc 1 mg/mL+ AgNO_3_ 0.5 μg/mL treatment compared to SrAc 1 mg/mL alone (Figure 1A,B). As there was no indication that this improved wound healing was due to improved cellular attachment, or cell spreading (based on the unchanged expression levels of vinculin, a master regulator, Figure S1), we evaluated the cell viability and measured the cellular metabolic activity upon chemical treatments. Figure 1C shows metabolic activity levels of HGFs after 24 h exposure to chemicals. Although some fluctuations in metabolic activity could be observed, no statistically significant differences were detected in different groups in comparison to the control group. Thus, the results suggest that the observed enhancement in cell migration is not a consequence of increased metabolic activity or cell growth.

### Effect of SrAc and AgNO_3_ on MSBF

**Figure 2:**
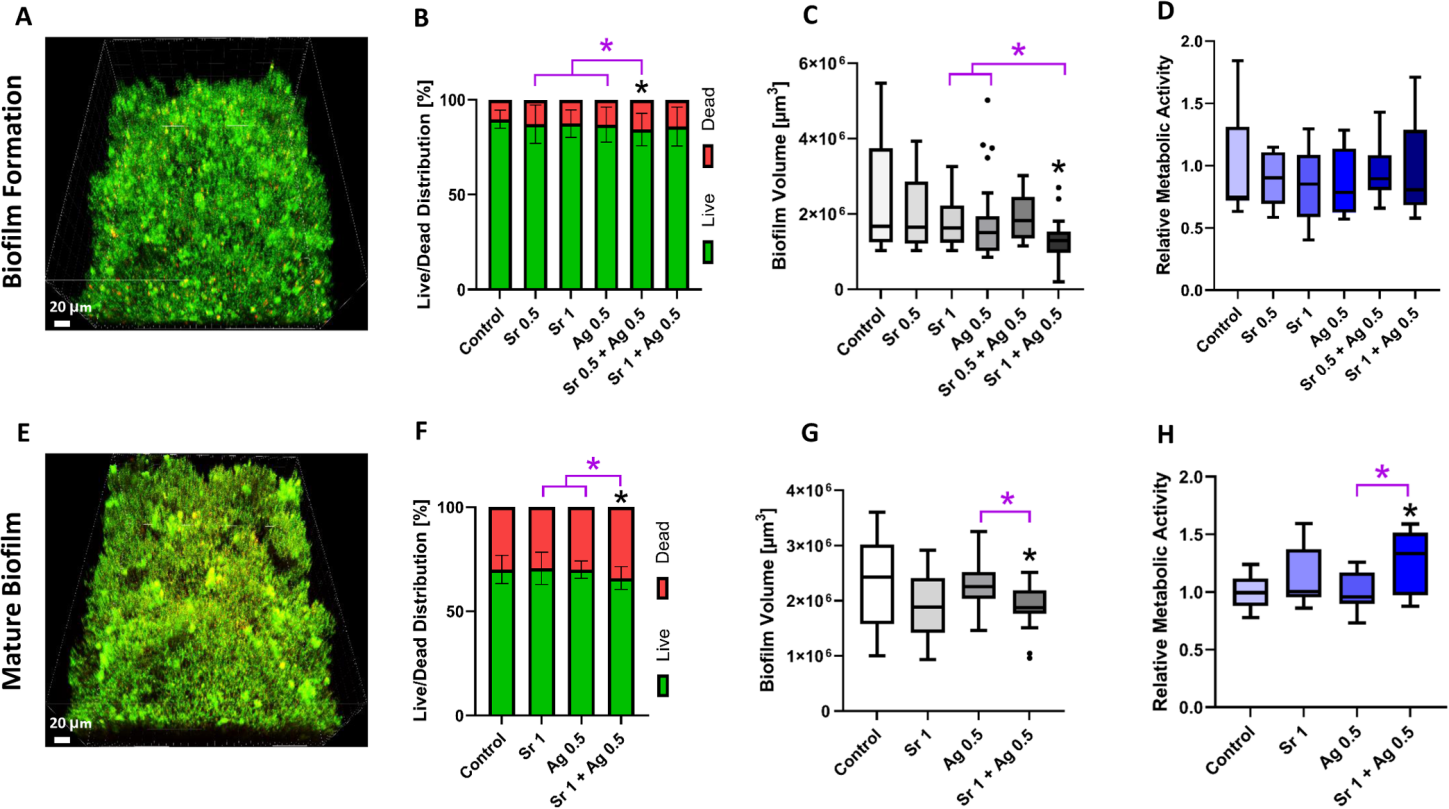
Effect of AgNO_3_ [μg/mL], SrAc [mg/mL], and their combination treatments on MSBF formation (0 h, simultaneous chemical treatment and biofilm formation process) after 24 h incubation (A–D) and early matured (24 h old) MSBF after 48 h incubation (E–H). (A, E) Representative 3D-reconstructed CLSM images of biofilms. Scale bars represent 20 μm. (B, F) Mean value ± standard deviation of the live/dead distribution. (C, G) Tukey box plots of total biofilm volume. (D, H) Tukey box plots of metabolic activity. Black stars indicate statistically significant difference compared to the control with *p* ≤ 0.05, whereas pink stars indicate statistically significant difference between connected groups. The differences of metabolic activity and live/dead distribution in early matured biofilm were assessed using one-way ANOVA test with multiple comparisons. To analyze the volume of MSBF in both biofilm formation and early matured biofilm, as well as the live/dead distribution in the 0 h biofilm, the Kruskal–Wallis test was applied. Data shown are representative of *N* = 9 independent experiments.

Using a set of four clinically relevant bacterial species, a comprehensive assessment of chemicals was conducted to examine their effects on biofilm formation, as well as on biofilm destruction. To investigate the effect of SrAc, AgNO_3_ and their combinations on MSBF formation, treatment started at 0 h (simultaneous chemical treatment and biofilm formation process) followed by 24 h incubation. The percentage of live/dead bacteria distribution (Figure 2B), biofilm volume (Figure 2C) and metabolic activity (Figure 2D) were monitored. Figure 2A shows the formed MSBF in the control group, which did not undergo any chemical treatment. The formation of this four-species biofilm was similarly observed in all groups. In terms of membrane integrity, the formed MSBF in the control group exhibited approximately 90% live bacteria and 10% (±4.8%) dead bacteria. As a result of adding chemicals simultaneously with the biofilm formation process, only the SrAc 0.5 mg/mL + AgNO_3_ 0.5 μg/mL group showed a statistically significant increase in dead cell percentage within biofilm compared to the control group (14.58 ± 6.3%). However, this combination had a stronger antibacterial effect on MSBF than each individual chemical at the same concentration (Figure 2B). The dead cell percentage of AgNO_3_ 0.5 μg/mL, SrAc 0.5 mg/mL, SrAc 1 mg/mL, and SrAc 1 mg/mL + AgNO_3_ 0.5 μg/mL was statistically equivalent to the control group. The average biofilm volume in the control group was 2.16 × 10^6^ μm^3^ (±1.37 × 10^6^ μm^3^) per image (Figure 2C). The quantified volume of the formed biofilm was found to be considerably lower only in the SrAc 1 mg/mL + AgNO_3_ 0.5 μg/mL group in comparison to the control group (Figure 2C). Similar to the live/dead distribution of bacteria, biofilm volume was reduced more in combination treatment (SrAc 1 mg/mL+ AgNO_3_ 0.5 μg/mL) than for each chemical alone. No significant differences were observed in the other groups. Finally, the metabolic activity of the biofilms was quantified by measuring the fluorescence intensity of resorufin. Bacterial metabolic activity was statistically equivalent in all groups (Figure 2D).

Based on the results on biofilm formation and cell migration, SrAc 1 mg/mL, AgNO_3_ 0.5 μg/mL and their combination were selected to evaluate their effects on early matured MSBF (treatment after 24 h precultivation) with an additional incubation period of 48 h, aiming to achieve comparable results with the coculture setup. Analyses were conducted similarly to those for biofilm formation (Figure 2E–H). The various morphologies of bacteria of the MSBF formed under the control conditions are shown in Figure 2E. A greater percentage of bacterial mortality was detected in all groups within the early matured biofilm following a 48 h incubation period, as compared to biofilm formation after 24 h (Figure 2F). Following a 48 h incubation period, the control group’s MSBF demonstrated a distribution of around 70% viable bacteria and 30% (±6.8%) dead bacteria. After 48 h incubation, the SrAc 1 mg/mL + AgNO_3_ 0.5 μg/mL group showed a slight but significant increase in the dead cell proportion in comparison to the control group (34 ± 5.4%). Notably, also in this experiment, the effect of exposure to the combination of the chemicals was stronger than separate treatments (Figure 2F). The control group exhibited an average biofilm volume of 2.31 × 10^6^ μm^3^ (±0.8 × 10^6^ μm^3^) per image. The biofilm volume in the SrAc 1 mg/mL group was lower (1.90 × 10^6^ μm^3^ ± 0.5 × 10^6^ μm^3^), although no statistically significant differences were identified between these two groups. In contrast, the combined treatment (SrAc 1 mg/mL + AgNO_3_ 0.5 μg/mL) resulted in a significant reduction in biofilm thickness (1.89 × 10^6^ μm^3^ ± 0.3 × 10^6^ μm^3^) (Figure 2G) as well as a significant, maybe stress-induced increase in metabolic activity (Figure 2H). These effects of the combined treatment on both biofilm volume and metabolic activity were also significantly different from those of the AgNO_3_ 0.5 μg/mL group. Accordingly, the biofilm volume was found to be lower, while the metabolic activity was higher in the combined treatment group. Thus, the combined treatment showed higher antibacterial activities and reduced biofilm volume compared with single treatments.

### Effect of SrAc and AgNO_3_ in the INTERbACT Model

**Figure 3:**
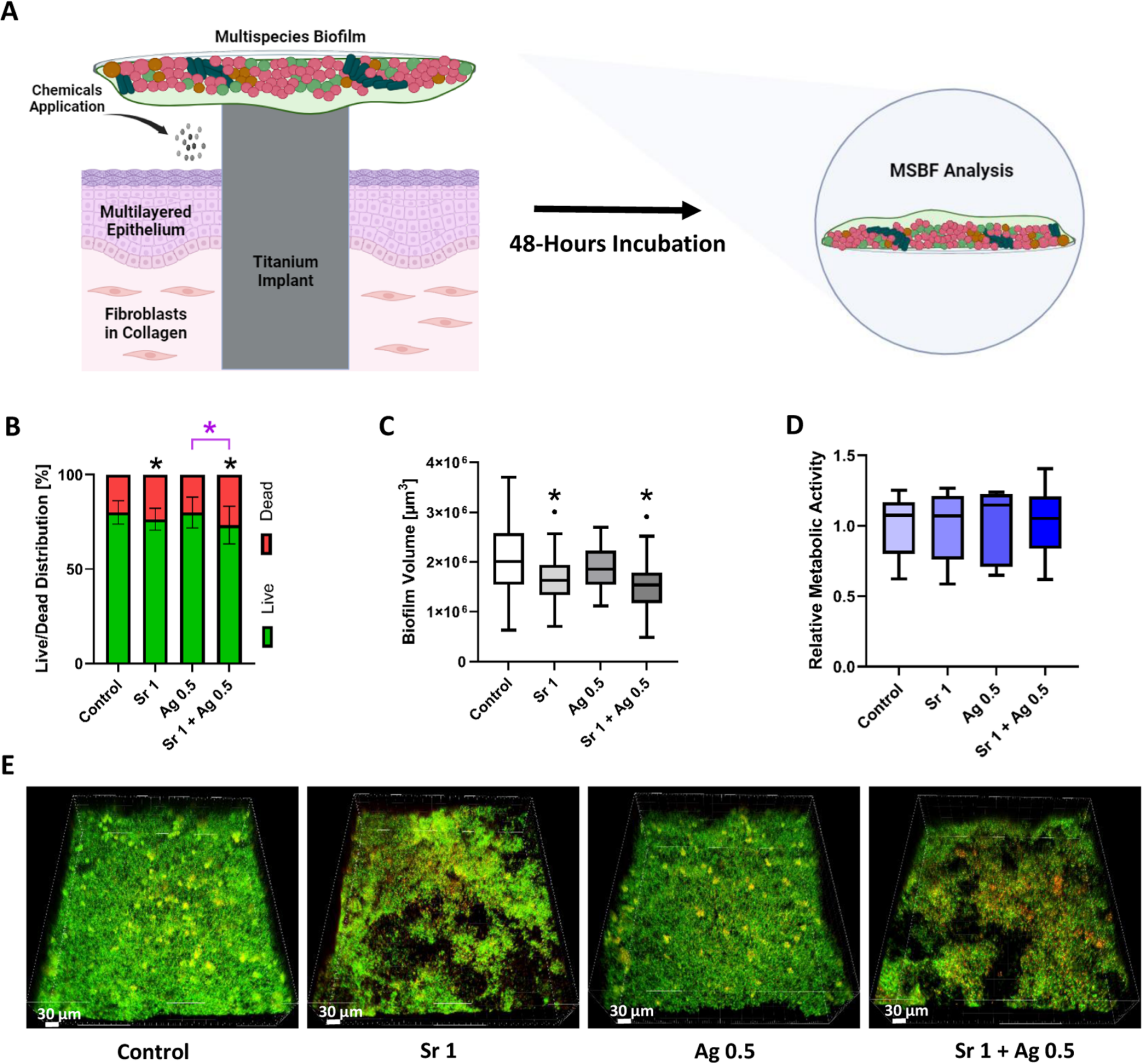
Effect of AgNO_3_ [μg/mL], SrAc [mg/mL] and their combination treatments on the 3D implant-tissue-oral bacterial-biofilm model (INTERbACT model): insights from biofilm analysis. (A) Schematic illustration of the INTERbACT model and experimental setting (created with BioRender.com). (B) Mean value ± standard deviation of the live/dead distribution of MSBF. (C) Tukey box plots of total biofilm volume. (D) Tukey box plots of MSBF’s metabolic activity. (E) Representative CLSM images of bacterial biofilms in each group. Scale bars represent 30 μm. Black stars indicate statistically significant difference compared to the control with *p* ≤ 0.05, whereas pink stars indicate statistically significant difference between connected groups. Statistical significance in biofilm volume, live/dead distribution, metabolic activity was assessed through one-way ANOVA test with multiple comparisons. Data shown are representative of *N* = 12 independent experiments.

Following the establishment of the early matured MSBF on cover glasses (24 h old) and parallel assembly of a three-dimensional artificial oral mucosa with integrated implant according to an established protocol,^29^ the coculture experiments were conducted in the INTERbACT model. For this purpose, the chemicals were added in the INTERbACT model and incubated for 48 h (Figure 3A). Afterward, biofilms were removed and analyzed (Figure 3B–E). The average percentage of dead bacteria in the control group was 19.90% (±6.2%), which was lower than in the monoculture setup (Figure S2). In contrast to the monoculture setup, the SrAc 1 mg/mL group exhibited a statistically significant rise in the proportion of dead cells compared to the control group (23.6 ± 5.8%). A significant increase in dead cells was also observed in the SrAc 1 mg/mL + AgNO_3_ 0.5 μg/mL group (26.7 ± 9.96%) compared to the control group and to AgNO_3_ 0.5 μg/mL, similarly to the monoculture setup (Figures 3B and S2). The average biofilm volume in the control group was 2.05 × 10^6^ μm^3^ (±0.7 × 10^6^ μm^3^). However, after chemical treatment, there was a significant reduction in volume in the SrAc 1 mg/mL (1.69 × 10^6^ μm^3^ ± 0.6 × 10^6^ μm^3^) and SrAc 1 mg/mL + AgNO_3_ 0.5 μg/mL (1.51 × 10^6^ μm^3^ ± 0.5 × 10^6^ μm^3^) groups. Figures 3E and S3 illustrate 3D reconstructions of the biofilms following a 48 h chemical treatment, depicting the partial decomposition and detachment of biofilm from cover glass in the SrAc 1 mg/mL and SrAc 1 mg/mL + AgNO_3_ 0.5 μg/mL groups, leading to a decreased biofilm thickness. Compared to their respective counterparts in monoculture conditions, in the 3D coculture model, the average biofilm volume exhibited reductions of 11.28, 12.1, 17.58, and 20.58% for the control, SrAc 1 mg/mL, AgNO_3_ 0.5 μg/mL, and SrAc 1 mg/mL + AgNO_3_ 0.5 μg/mL groups, respectively (Figure S2). Regarding metabolic activity, all groups demonstrated comparable levels in the 3D coculture model independent of treatment (Figure 3D).

**Figure 4:**
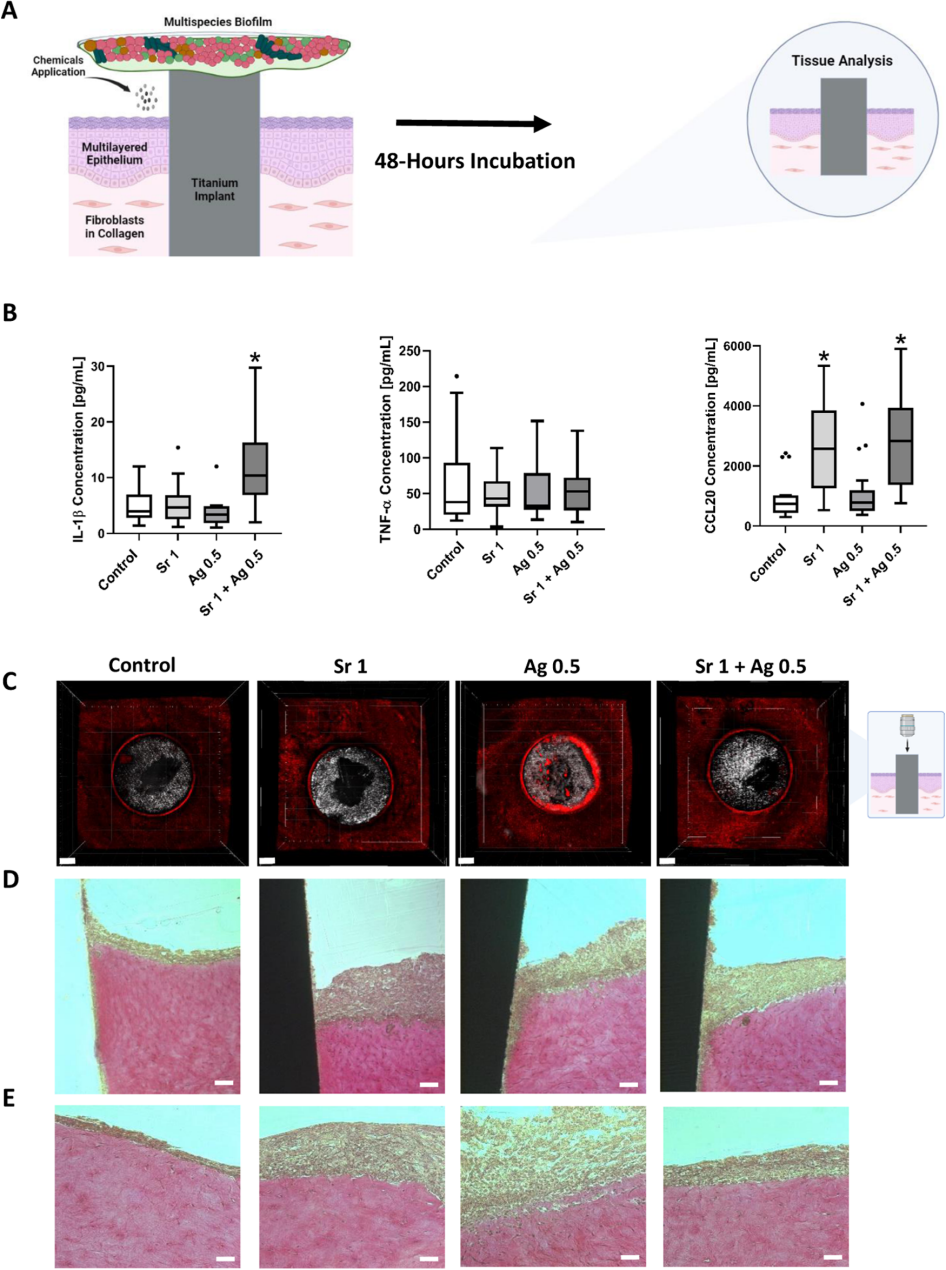
Effect of AgNO_3_ [μg/mL], SrAc [mg/mL], and their combination treatments on the 3D implant-tissue-oral bacterial-biofilm model (INTERbACT model): insights from tissue-related analysis. (A) Schematic illustration of the INTERbACT model and experimental setting (created with BioRender.com). (B) Tukey box plots depicting the secretion of cytokines and chemokines by peri-implant mucosa following 48 h exposure to MSBF and chemical agents and *N* = 12. (C) BacLight staining of the complete model after 48 h treatment with corresponding chemicals. The samples were examined under the CLSM. Scale bars represent 500 μm and *N* = 6. (D, E) Histological sections and van Giesson staining of the peri-implant mucosa after 48 h exposure with corresponding chemicals and MSBF. Implant-mucosa interface is shown in (D) and mucosa at a distance from implant is shown in (E). Scale bars represent 100 μm (D) and 50 μm (E) and *N* = 6. Black stars indicate statistically significant difference compared to the control with *p* ≤ 0.05, whereas pink stars indicate statistically significant difference between connected groups. The disparities in cytokine expressions were analyzed using the Kruskal–Wallis test. Scale bars represent 30 μm.

To analyze the response of tissue cells in the different conditions, the production of inflammation-associated cytokines IL-1β, TNF-α and the chemokine CCL20 by the human cells of the 3D peri-implant oral mucosa model were determined by ELISA assays of the supernatant (Figure 4A,B). After 48 h exposure to biofilm and chemicals, IL-1β secretion was significantly increased only in combined treatment of SrAc 1 mg/mL + AgNO_3_ 0.5 μg/mL in comparison to the control group (3D tissue exposure only to biofilm, without chemical exposure). This shows a unique effect of this combination on the inflammatory responses. AgNO_3_ 0.5 μg/mL and SrAc 1 mg/mL as individual treatments had no impact on the IL-1β level. The addition of chemicals did not change TNF-α secretion in any of the groups. In contrast, a significant increase in CCL20 secretion was observed in both groups containing SrAc (SrAc 1 mg/mL and SrAc 1 mg/mL + AgNO_3_ 0.5 μg/mL) compared to the control group, whereas the AgNO_3_ 0.5 μg/mL group did not show any significant difference in CCL20 levels.

Using live/dead staining and histological examinations, the impact of the biofilm and chemicals on the peri-implant cells and structure was investigated after 48 h (Figures 4C–E, S4, and S5). Among all of the stained tissues, the group treated with AgNO_3_ only exhibited an enhanced red color indicative of dead cells (Figure 4C). This increase was most prominent on the top layer cells, whereas the cells inside the tissue showed similar red and green fluorescence compared to the other groups (Figure S5). In the AgNO_3_-treated groups, disruption of the connective tissue–implant interface and apical migration of the epithelium along the implant surface were observed. Regarding histology results, after a 48 h biofilm challenge, the mucosa in the control group appeared thin, compact, and only with slightly loosened cell structure especially adjacent to the implant (Figure 4D,E). In both SrAc-treated groups, the epithelium showed an increased thickness and cellular loosening especially close to the implant, while still maintaining its structural integrity at more distance. In contrast, in the AgNO_3_-treated group, histology results demonstrated distortion and loosening of the epithelium in both the adjacent areas and the areas at a distance from the implant site. The integrity of underlying connective tissue was preserved in all groups, although slight detachment of epithelial tissue from connective tissue was observed in AgNO_3_ alone treated groups (Figure 4D,E). Overall, the combined SrAc/AgNO_3_ treatment exhibited an antibacterial effect while least compromising tissue structural integrity.

## Discussion

The clinical success of dental implants depends on achieving adequate tissue formation, including successful osseointegration and effective soft tissue seal around implant’s transmucosal area, while also preventing the formation of biofilms and infection. Inadequate healing of soft tissues around implant surfaces would allow bacterial infiltration toward the bone area, thereby hindering bone formation and causing inflammatory reactions leading to tissue destruction.^1,8,33^ It has been acknowledged that the soft tissue serves not only as a passive barrier, but also exhibits active responses to external stimuli through the production of different cytokines, chemokines, adhesion molecules, growth factors, and matrix metalloproteases, enabling it to effectively counteract microbial threats.^1,2^ Hence, there is an ongoing need to create innovative strategies on implant surfaces that promote healing of soft tissues and provide antibacterial effects as well. Despite the abundance of literature concerning the modification of peri-implant environment to enhance osseointegration and antibacterial properties, there is a lack of research on strategies to improve soft tissue healing at the mucosal tissue–implant interface and at the same time reduce bacterial infection.^33,34^ Therefore, the aim of this study was to thoroughly assess the antibacterial properties and soft tissue healing effects of AgNO_3_, SrAc, and their combination using complex testing models that closely mimic the situation in the oral cavity, thereby enhancing the translational relevance of the findings to clinical scenarios.

The creation of an effective soft tissue seal around implant surfaces relies on cellular interactions occurring at the interface between mucosal tissue and the implant surface.^35^ Upon implant insertion, the peri-implant connective tissue that develops during the healing process exhibits differences from the natural periodontium by having less vascularity, parallel collagen fibers, and a significantly low number of fibroblasts (66–80% less than healthy periodontal tissue), which contributes to decreased tissue healing, weaker seal and potential bacterial invasion subsequently.^8,36^ The migration, adhesion, and proliferation of HGFs as the dominant cells in peri-implant soft tissue are crucial for healing, extracellular matrix formation, remodeling, and immune responses against bacteria.^37^ Micro- or nanopatterned topographies, co-implanted magnesium/zinc coatings, and growth factor incorporation improved HGFs proliferation, migration, or adhesion suggesting possible soft tissue healing strategies.^38−40^ In the present study, our recently discovered optimal AgNO_3_ and SrAc concentration and their combinations, which previously showed osteogenic and synergistic antibacterial effects,^12^ were evaluated for their potential to heal soft tissues. For this purpose, first monolayer of HGFs were evaluated regarding migration and metabolic activity using standard monoculture assays.^32,41^ The results revealed cytocompatibility, significant enhancement in cell migration in all time points, and almost complete wound closure after 36 h with the addition of SrAc (0.5 and 1 mg/mL). Previous investigations have yielded similar results showing a promoted HGFs viability and migration and *in vitro* wound repair following treatment with Sr-citrate solution and released Sr from loaded alginate hydrogels.^19,21^ Moreover, Sr ions released from alginate hydrogels promoted angiogenesis, collagen deposition, proliferation, and migration of vascular endothelial cells and fibroblasts, together resulting in wound healing acceleration both *in vitro* and in an *in vivo* skin defect model.^42^ Regarding AgNO_3_, the absence of negative effects on cell migration and unaffected cellular viability supports the cytocompatibility at concentrations as applied in our study. However, the combination of AgNO_3_ 0.5 μg/mL and SrAc 1 mg/mL did not lead to wound closure, in contrast to the combination of AgNO_3_ 0.5 μg/mL with SrAc 0.5 mg/mL. This observation supports our prior findings on a critical threshold in the moderating effect of SrAc in combination with AgNO_3_.^12^ Moreover, AgNO_3_ may have introduced complex modulations of cellular signaling pathways or cell membrane properties that modify the response of cells to certain SrAc concentrations. Yamaguchi-Ueda et al. previously demonstrated that the combination of ions including Sr^2+^, boron (B), aluminum (Al^3+^), silicon (Si), and sodium (Na^+^) effectively induce the migration of HGFs by activating the extracellular signal-regulated kinase 1/2 (ERK1/2) signaling pathway which is relevant for cell proliferation, migration, differentiation, and death.^43^ The putative interactions of SrAc- and AgNO_3_-induced cellular pathways highlight the significance of their combined application and the need for careful consideration of their concentrations. Nevertheless, the detailed mechanism underlying this effect is still unknown, and should be determined in further studies in order to predict a knowledge-based concentration for therapeutic application.

Another significant factor causing peri-implant inflammation and tissue loss is bacterial infection and biofilm formation.^44^ Various surface modifications with antibacterial properties have been investigated for implant surfaces. However, these modifications are not yet clinical routine.^45^ In our previous study, we defined a therapeutic, antibacterial as well as cytocompatible, range for AgNO_3_ and SrAc and observed a synergistic antibacterial effect when these two elements were combined against the oral bacterial strain *Aggregatibacter actinomycetemcomintas*.^12^ However, the experimental setup used did not fully replicate the clinical situation, where multiple bacteria attach, aggregate, form biofilms, and further progress through maturation, which makes it difficult for antibacterial agents to penetrate and kill bacteria.^46^ Understanding the impact of chemicals on the early biofilm formation dynamics is important as it sets the stage for subsequent biofilm growth and its potential implications on tissue healing. Therefore, we have initially tested the effect of AgNO_3_, SrAc, and their combination on biofilm formation using an oral four-species MSBF model for more accurately reflecting the clinical scenario regarding an early commensal biofilm. The reliable biofilm growth of the bacteria *S. oralis*, *A. naeslundii*, *V. dispar*, and *P. gingivalis* has been previously demonstrated and the model has been already used for assessing the antibacterial activity of various substances and materials.^30,47,48^ The individual concentrations of AgNO_3_ and SrAc that had previously demonstrated significant antibacterial activity against a single oral bacterial species,^12^ did not exhibit similar effects in the MSBF model. Compared with the control group, individual chemicals had no significant effect on the growth of MSBF, its metabolic activities, or the live/dead distributions within biofilms. The observed differences were associated with MSBF’s inherent ability to withstand toxic substances due to the biofilm matrix and modified gene expression, therefore reducing the susceptibility to the antibacterial properties of Ag and Sr,^46,49^ Interestingly, the combination of AgNO_3_ and SrAc exhibited a significant decrease in viability of bacteria within MSBF and reduced biofilm volume compared to each chemical individually. Similar to our previous observation,^12^ this result implies a potential synergistic effect between AgNO_3_ and SrAc, which might be due to different antimicrobial mechanism of each chemical that reduces bacteria’s ability to counteract them.^50^ While little is currently known about the antibacterial mechanism of Sr, Alshammari et al. demonstrated that Sr-functionalized titanium surfaces exhibit notable bactericidal and bacteriostatic effects against both monospecies and multispecies biofilms.^22^ A review has highlighted the limited antimicrobial effects of Sr-functionalized titanium surfaces against *Staphylococcus aureus*, albeit not *Escherichia coli*.^50^ Nonetheless, Sr ions have shown antibacterial activity against both bacterial species when combined with other metal ions (such as Ag^+^).^50^ These results are in line with our study and highlight the promising antibacterial capacity of combining AgNO_3_ and SrAc, although the detailed mechanism should be analyzed in future studies. Additionally, although we observed a significant antibacterial effect, it was not highly pronounced. Similarly, Kommerein et al. investigated the antibacterial effects of two antibiotics, amoxicillin and metronidazole, individually and in combination, at two concentrations (14 and 140 μg/mL) on MSBF formation after 24 h of incubation, as in our initial experiment. Amoxicillin and metronidazole treatment at 14 μg/mL did not significantly affect the total biofilm volume, aligning with the limited effect size observed in our study. However, at the higher concentration of amoxicillin (140 μg/mL) alone and in combination with metronidazole (140 μg/mL), they significantly reduced biofilm volume and increased the proportion of dead bacteria to 50–70%.^30^ These findings highlight the need for further studies to enhance the effect size to a clinically relevant level. Moreover, studies have shown that the antibacterial efficacy of Ag nanoparticles is influenced by both the concentration and exposure time. However, most studies examined exposure times of less than 24 h.^14^ Similarly, a review on antibacterial coatings for orthopedic implants reported that in coatings with a metal ion release mechanism, prolonged exposure to metal ions resulted in a stronger bactericidal effect. However, while the antibacterial effect is more pronounced, extended metal ion release can also lead to continuous stimulation of surrounding tissues and cells, potentially causing cytotoxicity.^51^ Since the first 24–48 h are crucial for biofilm formation, preventing colonization is most effective when the antibacterial agent is released rapidly during the initial period.^51,52^ In the current study, we applied a localized chemical treatment at the implant site for 24–48 h, targeting both early biofilm formation and early matured biofilms while ensuring cytocompatibility.

While the outcomes of these experiments illustrate the impact of chemicals on “preventing” the formation of biofilms, we have also assessed their influence on “treating” an early matured biofilm. In clinical settings, biofilm maturation occurs in distinct stages, starting with early colonizers like streptococcal species.^53^ As biofilms mature, bacterial metabolic activity increases, leading to microcolony formation and extracellular matrix formation. Maturation of MSBF progresses further by coaggregation with late colonizers which increase the biofilm’s pathogenicity. Biofilms maturate and grow further resulting in sessile structures, which exhibit increased resistance to eradication.^52,53^ In this study, we utilized a four-species oral MSBF model including early and middle colonizers which is highly reproducible within 24–48 h without requiring nutritional supplements from saliva or serum, ensuring a controlled growth medium. Additionally, the species distribution closely resembles natural conditions, making it a valuable model for studying early commensal biofilms.^30^ Though it differs from pathogenic biofilms in peri-implantitis, which develop over extended periods and incorporate a more complex and virulent bacterial community.^52,54^ Interestingly, testing the chemicals on this early matured biofilm (24 h old biofilms) yielded similar results to those observed during biofilm formation; the combination of SrAc and AgNO_3_ substantially reduced the volume of the biofilm and the proportion of living bacteria when compared to the control group and to each chemical alone. Nonetheless, the effect was stronger for live/dead distribution and weaker for biofilm volume compared to biofilm formation, which could be due to differences in matrix maturation and metabolic processes. If we define biofilm detachment as the separation of the biofilm from the cover glass, biofilm decomposition as the breakdown of the biofilm matrix and its structure, and biofilm disintegration as the complete collapse of the biofilm, partial detachment and decomposition of the biofilm from the cover glass was observed subsequent to the application of SrAc which resulted in reduced biofilm volume. This further supports the hypothesis of Sr affecting the biofilm’s structure. O’Sullivan et al. previously observed an anticolonizing effect of Sr when evaluating Sr-substituted apatite surfaces.^55^ Similarly, after exposing *E. coli* and *S. aureus* to Sr-substituted tricalcium phosphate coatings, both lost their biofilm-like structure and exhibited morphological changes, highlighting the antiadhesion and antibiofilm properties of the Sr-based coatings.^56^ However, further evaluation is required to assess the mechanism of this action. Regarding quantification of the metabolic activity of MSBF, for biofilm formation, equal levels in all groups could be revealed, which can be attributed to the complex dynamics of MSBF metabolism and the variety of bacterial responses during biofilm formation. However, on early matured biofilms, the combined treatment exhibited a significant increase in metabolic activity compared to AgNO_3_ alone. As the combination treatment resulted in bacterial toxicity as visible from the live/dead staining, this effect can be explained as stress reaction following increasing intracellular ROS levels upon exposure to bactericidal compounds.^57^ In summary, these results showed that the synergistic combination of AgNO_3_ and SrAc has the potential for antibacterial activity even against inherently resistant multispecies bacterial biofilms. As this is an important prerequisite for the development of antibacterial implant functionalization, it highlights the need for experimental conditions that comprise both the bacterial biofilms and the relevant cell types, as provided by the 3D coculture model.

As mentioned before, complex and dynamic host–microbe interactions play a pivotal role in determining oral health and disease states and, thus, also in the context of implant integration.^26^ To mimic these oral host–microbe interactions and better understand how they probably influence the wound healing and antibiofilm effect of SrAc/AgNO_3_, we have tested the chemicals using the INTERbACT model. This model consists of an artificial 3D mucosa, composed of collagen-embedded fibroblasts covered by a stratified epithelium layer, with an integrated titanium implant that can be cocultured with the oral multispecies biofilm.^29^ Previously, it has been used to examine the early host–microbe interaction based on inflammatory responses, transcriptional activity, microbial shift, and tissue integrity.^31^ In general, in the current study, the coculture of biofilm and tissue exhibited a reduced percentage of dead bacteria within the biofilm and a decrease in biofilm volume compared to the monoculture condition. These findings are consistent with an earlier investigation that similarly observed a reduction in biofilm volume and dead cell percentages after 24 and 48 h incubation of tissue with MSBF, as compared to biofilm alone.^31^ This indicates that the presence of tissue influences MSBF behavior in a dual-modulatory mode of action, reducing bacterial mortality within biofilm accompanied by reduced biofilm volume. The effect might potentially arise from antibacterial peptides generated by the tissue resulting in changing bacterial distribution, function, and cellular stress response.^31,58^ Most importantly, the coculture setting also magnified the significance of the toxic effects exerted by combined AgNO_3_ and SrAc against the biofilm, when compared to the monoculture setup. Here, even SrAc alone exhibited a significant antibacterial efficacy, resulting in a lower biofilm volume and a higher proportion of dead bacteria. This observation suggests a complex interaction among SrAc, tissue, and MSBF, implying that the inherent tissue reaction upon bacterial challenge could enhance the antibacterial effects of specific agents. This effect may be due to the increased susceptibility of additionally stressed bacteria within the biofilm due to both the toxic effect of SrAc and the tissue’s defense mechanism. Interestingly, in parallel, evaluation of metabolic activity in the 3D coculture model showed equivalent levels across all groups. However, this might be attributed to limitations in the assay’s specificity in this experimental setup. The resazurin assay, which was used in this study to assess viability, measures overall metabolic activity, which includes both bacterial and nonbacterial cellular activities leading to an inaccurate representation of the antibacterial effect in a coculture setting. In future studies, the metabolic activity of bacteria in a coculture with other cells should be specified using more complex methods, such as measuring the expression of specific metabolic genes. To our knowledge, no study has investigated the antibacterial properties of Sr or Sr/Ag in coculture with MSBF and tissue so far. Moreover, little is currently known about the antibacterial mechanism of Sr, as only a limited number of published studies on the antibacterial applications of Sr are available.^59^ However, a similar beneficial effect of coculture conditions on the antibacterial efficacy could be observed in our own previous studies, both for SrAc/AgNO_3_ as well as for Ag-gold alloy nanoparticles, even though more straightforward coculture conditions were used.^12,60^ This is of great importance not only for the application of Sr/Ag as dual-functional implant modification but also for the analysis of future functionalizations in general. If the synergistic effect of tissue self-defense and antibacterial substance is valid also for other bacterial strains and substances as well as on a molecular level, bacteria/cell coculture experiments should be integrated into every material screening.

Besides the effect on MSBF, also the reaction of the tissue cells of the 3D coculture model upon biofilm and chemical treatment was analyzed. Although several studies used 3D oral tissue models to evaluate the inflammatory response to simulated infection, none of them has tested the effect of Ag or Sr on early innate immune response in the context of a complex interplay involving chemical factors, bacteria, and tissue.^26^ The cytokines/chemokines analyzed in the current study were selected based on their differential expression toward biofilm challenge during the development of the 3D coculture model.^31^ Given that all groups in the current study were exposed to MSBF, any enhancement in cytokine/chemokine secretion could be attributed to the presence of the chemicals. Most notably, all SrAc-treated groups exhibited higher CCL20 secretion, a chemokine known for its additional antimicrobial properties.^61^ CCL20 exerts its immune effects through binding to chemokine receptor 6 (CCR6), initiating the migration of various immune cell types, including immature dendritic cells, B-cells, and T-cells. Beyond its role in receptor-mediated inflammatory responses, CCL20 possesses a unique feature due to its positively charged surface region. This characteristic allows CCL20 to function similarly to cationic antimicrobial peptides (AMPs), leading to bacterial membrane disruption and direct antibacterial effects.^61^ Therefore, additional antibacterial factors (e.g., antibacterial proteins/peptides, ROS) might contribute to the enhanced antibacterial effect in the coculture setup. This further supports the already described synergistic effect of SrAc/AgNO_3_ combination that not only affected antibacterial and cytocompatible properties but also enhanced early innate immune response. In contrast, secretion of the proinflammatory cytokine TNF-α did not change in response to chemical treatments, whereas secretion of IL-1β was enhanced only in the SrAc/AgNO_3_ combination group. A study by Choi et al. examined the immune-inflammatory and osteogenic impacts of Sr-incorporated titanium coatings.^62^ Their findings indicated that Sr-based surface modifications could potentially lead to the suppression of TNF-α and the upregulation of IL-10 secreted by a macrophage cell line. This effect, in turn, created a microenvironment that favored early wound healing associated with osteogenesis.^62^ Accordingly, Buache et al. exposed monocytes to lipopolysaccharides (LPS) to evaluate the effect of Sr-substituted biphasic calcium phosphate on the inflammatory response. The presence of Sr ions leads to decreased production of TNF-α but not of IL-1β.^63^ While these results collectively indicate that Sr has a potentially positive impact on the immune response, it is crucial to note that tissue-specific immunity differs from the inflammatory response of immune cells, making *in vivo* studies inevitable to reliably assess the immunogenic potential of Ag and Sr treatment.

Histological sections and live and dead stained tissues were used to visualize tissue morphology and cytotoxicity after exposure to MSBF with or without chemical treatment. In the control group, a slight loosening in the epithelial barrier of tissues, especially at the tissue–implant interface, was in alignment with previous investigation following 48 h exposure to biofilm.^31^ The contributing factors for this observation are most probably the downregulation of genes and signaling pathways related to cell adhesion, like cadherins, that would allow immune cells to migrate toward the bacterial biofilm.^30,64,65^ This reaction is, thus, not considered a pathologic effect of bacteria but a protective mechanism of the tissue itself. In tissues infected and treated with SrAc, the histology of the epithelial layer differed from the control group, showing increased thickness. In previous studies, changes in the thickness of epithelium were correlated to the rate of cell apoptosis suggesting histological appearance as analysis assay for toxicity evaluations.^66,67^ However, the increased thickness of the epithelium could also be interpreted as a sign of more progressive tissue loosening, possibly due to Sr-induced cytokine/chemokine secretion. Prior research indicated that excessive production of cytokines and chemokines leads to mucosal inflammation and tissue damage.^68^ However, these effects have not been studied in an *in vitro* model missing immune cells and thus lacking a full immune response. Hence, further research is needed to clarify the change in the morphology of the epithelium layer following SrAc treatment. In contrast, AgNO_3_ alone treated groups, which did not show any change in the expression of the selected cytokines, clearly exhibited a significant disruption in histological epithelium integrity and increased cytotoxicity, making them the most impactful condition in terms of tissue damage. Similarly, measuring transepithelial electrical resistance (TEER) on an intestinal epithelial model revealed that the barrier integrity was compromised following exposure to a nontoxic concentration of AgNO_3_ for 24 h.^69^ The apical migration of the epithelium along the implant surface in the AgNO_3_-treated group further confirmed cytotoxicity and impaired wound healing in this group. Animal studies have shown that disruption of the connective tissue–implant interface leads to undesirable apical migration of epithelium, which can negatively impact the implant’s primary stability.^70^ However, the epithelium in the SrAc/AgNO_3_ treatment group showed again better continuity that was comparable to that of the SrAc-treated group. This confirms our previous findings, which highlighted a reduction in cell toxicity of AgNO_3_ after combining it with SrAc.^12^ Therefore, application of combined SrAc/AgNO_3_ in a 3D coculture model not only reduced the cytotoxicity of single AgNO_3_ treatment but also enhanced epithelial continuity, affirming the positive impact of applying SrAc/AgNO_3_ for improved tissue integration. These findings set the promising basis for further research toward a clinical application of combined SrAc/AgNO_3_ treatment, aiming for enhanced tissue integration and antibacterial effect, and therefore longer-lasting implant success. However, as this is an *in vitro* study, it cannot fully replicate *in vivo* conditions, with limitations regarding incubation time, flow dynamics, immune cell responses, and other physiological factors.

## Conclusions

The findings of the present study clearly highlight the promising dual antibacterial and soft-tissue-integrative effect of a combined SrAc/AgNO_3_ treatment at noncytotoxic concentrations within the peri-dental implant environment during the initial healing phase, while considering the naturally occurring interactions between tissue and bacteria. A synergistic antibacterial effect was observed against early mature MSBF and during biofilm formation when SrAc and AgNO_3_ were used in combination. Moreover, a dual effect following SrAc application including improved cell migration and antibacterial activity could be shown, likely due to its impact on soft tissue inflammatory response. Besides these promising characteristics toward the clinical application of these chemicals, the results also underscore the significance of mimicking natural tissue–bacteria interactions in experimental setups for reliable *in vitro* testing of novel implant functionalization strategies that should be taken into account on a more general basis.

## Supporting Information Available

Effect of chemicals on expression of vinculin in HGFs; comparison of biofilm volume and proportion of dead bacteria in mature MSBF in monoculture and 3D coculture model; representative CLSM images of bacterial biofilms in each group within the INTERbACT model; representative CLSM images (top view) of the INTERbACT model following chemical treatments; and representative CLSM images (side view) of the INTERbACT model following chemical treatments (PDF)
