# Supplementary material for: Dual Antibacterial and Soft-Tissue-Integrative Effect of Combined Strontium Acetate and Silver Nitrate on Peri-Implant Environment: Insights from Multispecies Biofilms and a 3D Coculture Model
Source: ACS Appl Mater Interfaces. 2025 Apr 22;17(18):26282–95. doi: 10.1021/acsami.5c01093 (PMC12067368; doi:10.1021/acsami.5c01093)
Supplement: Supplementary file 1 — am5c01093_si_001.pdf [file am5c01093_si_001.pdf]

Supporting Information

Dual Antibacterial and Soft Tissue-Integrative Effect of Combined Strontium Acetate and Silver Nitrate on Peri-Implant Environment: Insights from Multispecies Biofilms and a 3D Co-Culture Model

Marjan Kheirmand-Parizi [1,2], Katharina Doll-Nikutta [1,2], Carina Mikolai [1,2], Dagmar Wirth [3], Henning Menzel [4], Meike Stiesch [1,2,]*

1- Department of Prosthetic Dentistry and Biomedical Materials Science, Hannover Medical School, Carl-Neuberg-Strasse 1, 30625 Hannover, Germany

2- Lower Saxony Center for Biomedical Engineering, Implant Research and Development (NIFE), Stadtfelddamm 34, 30625 Hannover, Germany

3- Helmholtz Centre for Infection Research, 38124, Braunschweig, Germany

4- Institute for Technical Chemistry, Braunschweig University of Technology, Hagenring 30, 38106 Braunschweig, Germany

Corresponding author: Stiesch.Meike@mh-hannover.de *

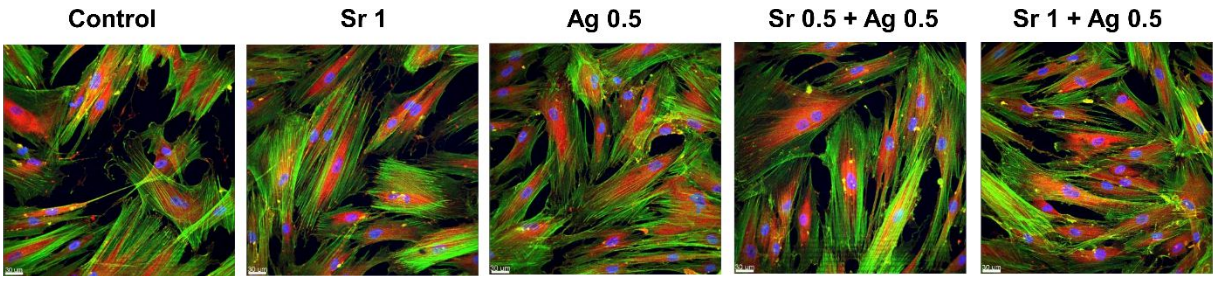

Figure S1. The effect of $AgNO_3$ [µg/mL], SrAc [mg/mL] and their combination treatments on expression of vinculin in HGFs. One-shot immunofluorescence staining of vinculin, a protein

of focal adhesion plaques (red) upon chemical treatment for 24 hours. The actin cytoskeleton is stained in green with phalloidin-iFluor 488, and the cell nuclei are stained in blue with DAPI. Scale bars represent 30 µm.

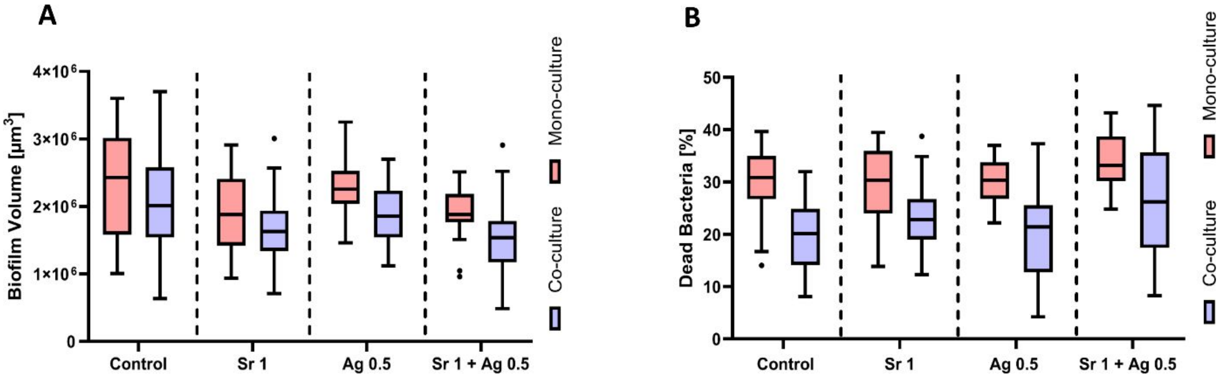

Figure S2. Comparison of biofilm volume (A) and proportion of dead bacteria (B) in mature (24 hours old) MSBF after 48 hours incubation in mono-culture and 3D co-culture model under various treatments $AgNO_3$ [µg/mL] and SrAc [mg/mL] and untreated conditions.

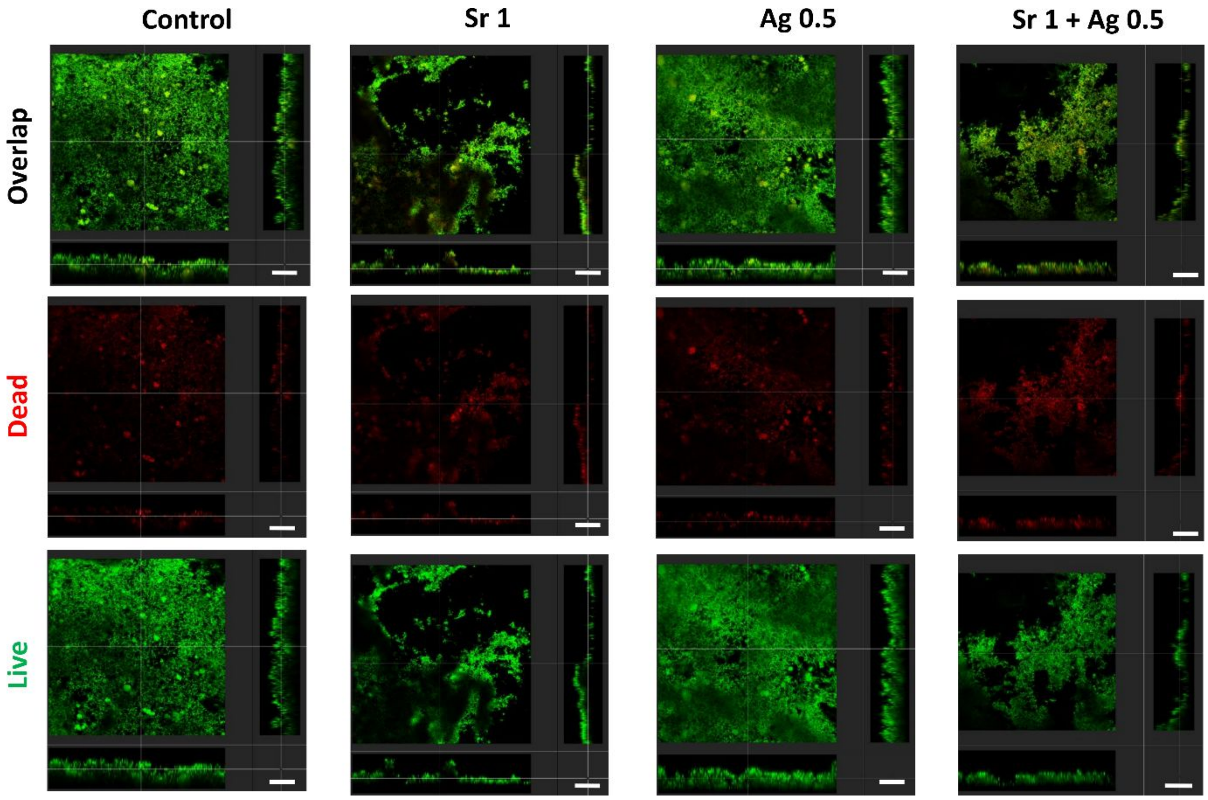

Figure S3. Representative CLSM images of bacterial biofilms in each group ($AgNO_3$ [μg/mL] and SrAc [mg/mL]) as well as the untreated condition within the 3D implant-tissue-oral bacterial-biofilm model (INTERbACT model). In cross-sectional views, the base of biofilm is oriented upwards. CLSM images show live bacteria stained green with SYTO-9 and dead bacteria stained red with propidium iodide (PI). Scale bars represent 30 μm.

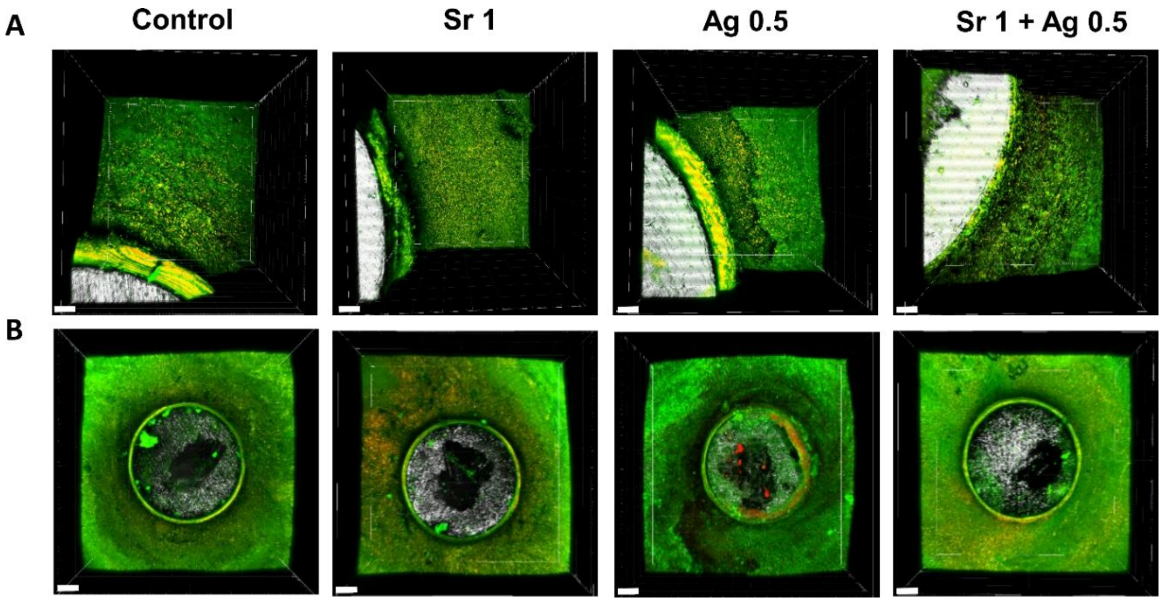

Figure S4. The effect of $AgNO_3$ [μg/mL], SrAc [mg/mL] and their combination treatments on the 3D implant-tissue-oral bacterial-biofilm model (INTERbACT model): insights from tissue-related analysis. (A, B) BacLight staining of the complete model after 48 hours treatment with corresponding chemicals. The samples were examined under the CLSM. Images were taken from top view. Titanium implants were visualized using reflection in CLSM. SYTO9, green; PI, red; implant, grey. Scale bars represent (A) 200μm, (B) 500μm.

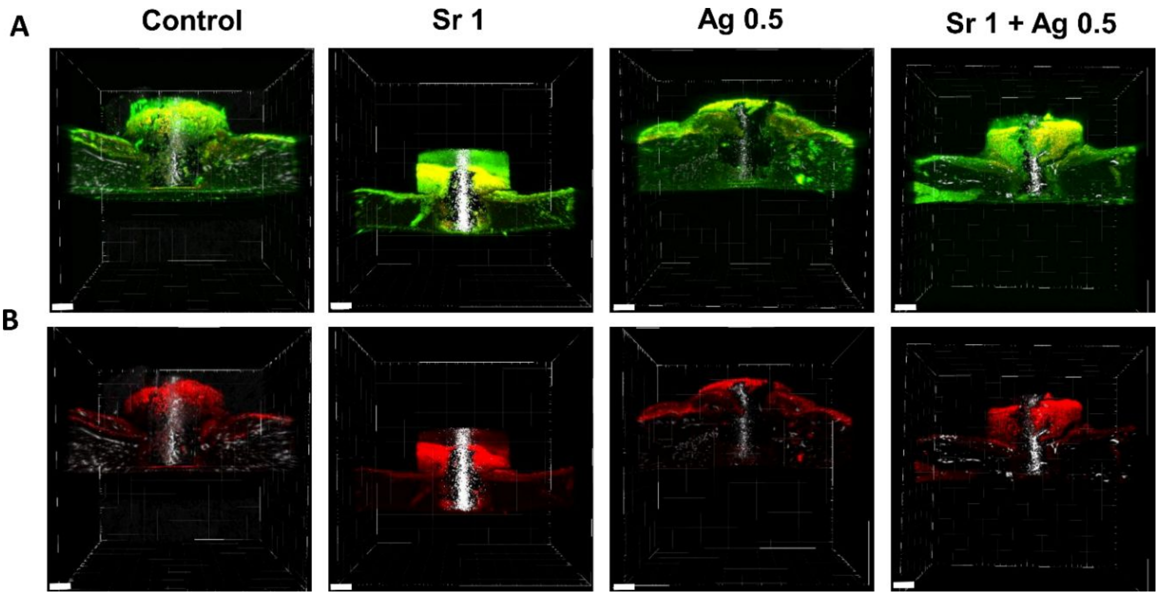

Figure S5. The effect of $AgNO_3$ [μg/mL], SrAc [mg/mL] and their combination treatments on the 3D implant-tissue-oral bacterial-biofilm model (INTERbACT model): insights from tissue-related analysis. BacLight staining of the complete model after 48 hours treatment with corresponding chemicals. (A) Displays an overlay of CLSM images using SYTO-9 (green) and PI staining (red), and (B) shows CLSM images stained with PI. Images were taken from side view. Titanium implants were visualized using reflection in CLSM (grey). Scale bars represent 500μm.
